# Imputation of sequence level genotypes in the Franches-Montagnes horse breed

**DOI:** 10.1186/s12711-014-0063-7

**Published:** 2014-10-01

**Authors:** Mirjam Frischknecht, Markus Neuditschko, Vidhya Jagannathan, Cord Drögemüller, Jens Tetens, Georg Thaller, Tosso Leeb, Stefan Rieder

**Affiliations:** Agroscope - Swiss National Stud Farm, 1580 Avenches, Switzerland; Institute of Genetics, Vetsuisse Faculty, University of Bern, 3001 Bern, Switzerland; Swiss Competence Center of Animal Breeding and Genetics, University of Bern, Bern University of Applied Sciences HAFL & Agroscope, 3001 Bern, Switzerland; Graduate School for Cellular and Molecular Biology, University of Bern, 3012 Bern, Switzerland; Institute of Animal Breeding and Husbandry, Christian-Albrechts-University, 24118 Kiel, Germany

## Abstract

**Background:**

A cost-effective strategy to increase the density of available markers within a population is to sequence a small proportion of the population and impute whole-genome sequence data for the remaining population. Increased densities of typed markers are advantageous for genome-wide association studies (GWAS) and genomic predictions.

**Methods:**

We obtained genotypes for 54 602 SNPs (single nucleotide polymorphisms) in 1077 Franches-Montagnes (FM) horses and Illumina paired-end whole-genome sequencing data for 30 FM horses and 14 Warmblood horses. After variant calling, the sequence-derived SNP genotypes (~13 million SNPs) were used for genotype imputation with the software programs Beagle, Impute2 and FImpute.

**Results:**

The mean imputation accuracy of FM horses using Impute2 was 92.0%. Imputation accuracy using Beagle and FImpute was 74.3% and 77.2%, respectively. In addition, for Impute2 we determined the imputation accuracy of all individual horses in the validation population, which ranged from 85.7% to 99.8%. The subsequent inclusion of Warmblood sequence data further increased the correlation between true and imputed genotypes for most horses, especially for horses with a high level of admixture. The final imputation accuracy of the horses ranged from 91.2% to 99.5%.

**Conclusions:**

Using Impute2, the imputation accuracy was higher than 91% for all horses in the validation population, which indicates that direct imputation of 50k SNP-chip data to sequence level genotypes is feasible in the FM population. The individual imputation accuracy depended mainly on the applied software and the level of admixture.

**Electronic supplementary material:**

The online version of this article (doi:10.1186/s12711-014-0063-7) contains supplementary material, which is available to authorized users.

## Background

Rapid innovations in high-throughput sequencing and array technologies have drastically reduced the costs of next-generation sequencing (NGS) [1], which has made it feasible to re-sequence a large fraction of any mammalian genome. However, sequencing thousands of individuals is still too costly for routine implementation in breeding programs. To date, 50 k SNP (single nucleotide polymorphism)-chips typically build the genetic resource for genomic predictions and genome-wide association studies (GWAS) in livestock and other species [2]. With SNP-chips, thousands of individuals can be cost-effectively genotyped. However, depending on the extent of linkage disequilibrium (LD) in a given population, it has been estimated that a reasonably powered GWAS requires as many as 300 000 to 500 000 SNPs [3,4]. In cattle, a high-density (HD) SNP-chip was developed that contains 777 k SNPs [5], but HD SNP-chips are not yet available for most other livestock animals. However compared to NGS data, HD SNP-chips only represent a small fraction of the variation, given the 17 million DNA variants that were determined in cattle [6].

NGS thus represents a powerful alternative to array-based genotyping methods. To circumvent the economical and logistical difficulties involved in re-sequencing more than 1000 individuals, genotype imputation can be performed. Genotype imputation is a well-established method to combine information across collections of individuals with similar ancestry [7,8] and to derive HD genotype information for individuals that were genotyped on a low or medium density set of loci [9-13]. In livestock, genotype imputation accuracies have been mainly investigated in cattle, imputing low-density (3 k and 6 k) to medium (50 k) and medium to HD (777 k) SNP panels. The reported genotype imputation accuracies obtained in these studies ranged from 91.2% for imputation from 3 k to 50 k [11], to 99.1% from 6 k to 50 k [13] and to 99.7% from 50 k to 777 k [14]. Results in cattle show that the imputation of high-quality genotypes strongly depends on diverse parameters, including the proportion of missing genotypes [10], the effective population size (N_e_), the level of LD [14], the number of key ancestors and relatives in the reference population [9], and the imputation algorithm applied [10,11]. Besides cattle, genotype imputation has also been investigated in pig [15], sheep [12] and horse [7]. So far, in horse, imputation has been performed to combine 50 k and 65 k genotypes of various horse breeds. Imputation accuracies ranged from 82.2% to 100% [7].

Here, we investigated the accuracy of direct imputation from 50 k SNP-chip data to sequence-level genotypes in the Franches-Montagnes (FM) horse breed. The FM breed is the last indigenous Swiss horse breed [16,17]. In the past, this breed was particularly used as a working horse in agriculture and transport. Nowadays, the main purpose of the breed includes leisure riding and driving activities [16,17]. During their breed history, FM horses have been crossbred and thus systematically admixed (e.g. with Warmblood and Arabians) to enhance their gait and riding ability. The last introgression with two Warmblood stallions occurred in the 1990s and is represented today by the stallion lineages N and Q in the studbook [18]. Shortly after this introgression, the studbook of the FM breed was closed. Estimates of the current N_e_ of the FM breed ranges from 29.1 to 128.1, depending on the methodology applied [19].

We have successfully identified major quantitative trait loci (QTL) for height and maxillary prognathism in FM horses [20,21]. However, we could not detect genome-wide associations for many other traits. Insufficient marker density of the applied SNP-chip (50 k) could be a possible reason. Increasing the marker density has been demonstrated to improve the power of genomic prediction [10] and GWAS [6]. Therefore, the objectives of this study were to evaluate three methods for genotype imputation from the 50 k SNP-chip data to sequence level in the FM population.

## Methods

### Animals

We genotyped 1077 horses of the FM horse breed with the Illumina Equine SNP50 BeadChip® that includes 54 602 SNPs. This dataset has been previously described in detail [20]. We then selected 20 highly informative FM horses based on Principal Component Analysis (PCA) information scores [22] for whole-genome sequencing. In addition, we selected a few influential ancestors and progeny of these horses to increase the phasing accuracy. In total, 30 FM horses including two trios (sire, dam and offspring) and three duos (one parent and offspring) were sequenced. From this dataset, 28 FM horses were already included in the aforementioned dataset of 1077 genotyped FM horses, while the other two horses were additionally genotyped on the 65 k SNP-chip, which shares 40 000 SNPs with the 50 k SNP-chip.

The pedigree information of the 1079 horses revealed that the sires and the dams were included in the genotyped dataset for 707 and 207 horses, respectively. We also used pedigree information to determine the proportion of admixture by calculating the pedigree-based relatedness with crossbred horses. FM horses have experienced introgressions in the past, especially with Warmblood. In total, 11 of the 30 sequenced horses showed a level of admixture greater than 10%. To account for the effect of admixture, we included 14 unrelated Warmblood horses, for which NGS data was available, in our analyses. Thus, altogether, a total of 44 horses, including 30 FM horses and 14 unrelated Warmblood horses were included in the analyses.

### Next-generation sequencing and variant calling

We prepared fragment libraries with 300 bp insert size and collected one lane of Illumina HiSeq2000 paired-end reads (2 × 100 bp) for each horse.

The fastq sequence reads were subjected to initial quality checks (average read length, average read quality, average read quality per position, distribution of bases along the sequence length) using FastQC [23]. Sequences of average length of 100 nucleotides were aligned against the reference genome EquCab2.0 using the Burrows-Wheeler Alignment tool (BWA) version 0.5.9. [24] with default parameters. The aligned data were processed with SAMtools [25] and picard [26] to sort them by chromosome coordinates and to mark duplicates. The Genome Analysis Toolkit (GATK) [27] was used for indel realignment, SNP calling, and SNP filtering. Reads marked as duplicates and with a mapping quality less than 30 were excluded for variant calling. Raw variant data in variant call format (version 4.0) were flagged for low quality and unreliable variants using the variant filtration module of GATK. Variant filtration was defined according to the GATK recommended best practices documentation [28]. Analysis parameters like variant confidence (from the QUAL field) divided by the unfiltered depth of non-reference samples, Fishers exact test to detect strand bias, HaplotypeScore, Ranksum test score for read mapping quality and the distance of the allele from the end of the read were used to filter SNPs as specified in the document. SNPs that did not match any of these conditions were considered good and marked PASS in the output VCF file.

### Genotype concordance of sequenced horses

We compared the array-derived genotypes with the sequence-derived genotypes for the 30 sequenced FM horses. Genotype concordance of sequenced horses is defined as the ratio of identical genotypes and the total number of common SNPs typed with both methods. We had two horses with less than 95% genotype concordance, which were excluded from further analysis.

### Genotype imputation

We compared three commonly used imputation programs, including two population-based methods, Beagle [29] and Impute2 [30], and one method that combines LD and pedigree information, FImpute [31]. Methods were evaluated for two equine chromosomes (ECA), ECA16 and ECA31. All programs were run with default parameters, except where noted.

The imputation software package Beagle uses a so-called “localized haplotype-cluster model” to reconstruct haplotypes that are present in the reference population and a hidden Markov model (HMM) to calculate missing genotypes. In order to investigate the impact of different parameter settings using Beagle, we performed three imputation scenarios based on (i) un-phased genotypes, (ii) pre-phased genotypes (pre-phasing also with Beagle) and (iii) pre-phased genotypes including information of duos and trios of the reference population.

Impute2 is also based on a HMM, but reconstructs haplotypes that are present in the reference and test population. For the final genotype imputation, the haplotype structure of the reference population is used. In addition, we used the program SHAPEIT [32] to pre-phase the genotypes of the data, since this program also includes first-generation family information (duos and trios) for haplotype reconstruction. We set N_e_ equal to 100 for SHAPEIT and Impute2, which is a reliable estimate of the current N_e_ of the FM breed [19]. In Impute2, imputation was performed for fragments of 6 Mb. Output files of Impute2 were converted into ped and map files using GTOOL v0.7.5 [33]. GTOOL by default only converts SNP genotypes that have a genotype probability greater than 0.9, while genotypes below this threshold are set as missing. To provide an overall comparison between the three applied imputation methods, we set this threshold to 0, such that all genotypes were called. For all other analyses the default setting was used.

The third method that we used was FImpute, which reconstructs haplotypes using family and pedigree information and performs imputation based on haplotype consistency of overlapping sliding windows [31].

### Accuracy of imputation methods

We applied a cross-validation scheme to measure the accuracy of genotype imputation. Accuracy was defined as genotype concordance between the genotypes from NGS and the genotypes obtained by imputation. We had 28 FM horses with genome sequence and either 50 k SNP-chip genotypes (n = 26) or 65 k SNP-chip genotypes (n = 2). We split these horses into a test population of four horses and a reference population of 24 horses. We repeated this procedure seven times, such that each horse with genome sequence and 50 k SNP-chip genotypes was represented once in the test group. The horses with 65 k SNP-chip information were only used in the reference group. The duos and trios were equally distributed over all groups. We included all 1077 FM horses with 50 k SNP-chip genotypes for the haplotype reconstruction. Imputation from 50 k to sequence level genotypes was performed seven times, each time using a different group as test and reference population.

After imputation, we determined the genotype concordance rate between true and imputed genotypes of the 26 sequenced horses, to evaluate the accuracy of imputation. For Beagle and Impute2, accuracy was assessed using the commands merge and merge-mode 7, as implemented in Plink [34]. For FImpute an R-code [35] was applied. Using Plink, the concordance genotype rate is calculated based on the ratio of identical genotypes between sequenced and imputed loci, while genotype errors based on allelic state (homozygous vs. heterozygous) and origin (homozygous reference vs. homozygous alternative) are not differentiated.

After the evaluation of the three imputation methods, all chromosomes were imputed with Impute2. Accuracy was assessed for each chromosome by the same cross-validation scheme as described above. Furthermore, we calculated the ratio between correctly imputed SNP genotypes and the number of non-missing genotypes for each chromosome using the “diff” and “lmiss” files implemented in Plink.

## Results

### Whole-genome sequencing and SNP calling of the 44 horse genomes

We sequenced the genomes of 30 FM horses and 14 Warmblood horses. On average, we obtained 384 767 951 reads per animal, of which 94% mapped to the reference genome Equcab2.0. On average, there were 348 621 822 uniquely mapped reads per horse. The depth of coverage ranged from 2.66x to 25.27x [See Additional file 1]. On average, 8.66% of the mapped reads were marked as duplicate reads that aligned with identical start and end positions on the reference genome. We then called SNPs of the sequenced horses with respect to the reference genome and used 13 127 080 informative SNPs with a minor allelic frequency (MAF) greater than 1.5% and marked PASS for subsequent genotype imputation analyses.

### Genotype concordance of the 30 sequenced FM horses

**Figure 1:**
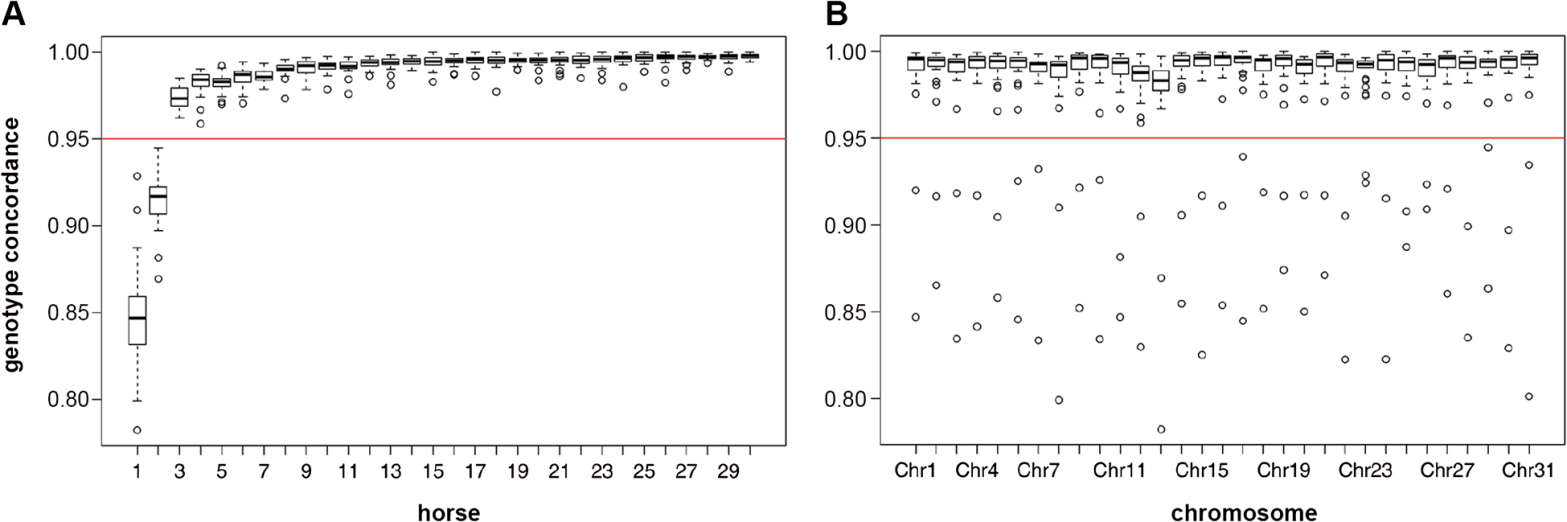
**Concordance between sequence-derived and SNP-chip-derived genotypes. (A)** Concordance per horse: the boxplots illustrate the distribution of the values for each chromosome within each of the 30 sequenced FM horses; two horses that had genotype concordances smaller than 0.95 were excluded from subsequent analyses. **(B)** Concordance per chromosome: the boxplots illustrate the values for each horse within each autosome; note that there are two outliers for each chromosome that are below the threshold of 0.95; these two outliers correspond to the two horses that were subsequently excluded.

We compared the concordance between SNP-chip-derived genotypes and sequence-derived genotypes as a quality control. The mean overall concordance per horse was 98.5% (Figure 1A). The genotype concordance of two horses was very low compared to all other horses, with 85 and 91%, respectively. These two horses also had the lowest sequence coverage, which is the most likely explanation for the low genotyping concordance. They were excluded from further analyses. The genotype concordance for each of the remaining 28 FM horses was greater than 97%.

We also analyzed the genotype concordance per chromosome (Figure 1B). ECA8, ECA12, and ECA13 had concordance values between 98 and 99%. All other chromosomes had a mean concordance greater than 99% across all horses. After these quality control steps, we retained 42 sequenced horses and 1077 horses with SNP-chip genotypes for the final genotype imputation.

### Genotype imputation accuracy of three software programs

We imputed all SNPs of ECA16 and ECA31 for the sequenced horses in a cross-validation experiment. In this experiment, we had a total of 26 FM horses with both 50 k SNP-chip data and whole-genome sequence data. We analyzed the accuracy of imputation using three software programs (Table 1). With all three programs, we found only very small differences in accuracy between the two chromosomes. Impute2 outperformed the two other programs and yielded a mean accuracy of ~92%. The two other programs had accuracies of ~77% (FImpute) and ~75% (Beagle). FImpute was computationally the most efficient program, yielding results within 15 minutes for ECA16, which included 470 000 SNPs, on an Intel Core2, 2.8 GHz CPU with 98 GB of RAM. Running time increased to 6 and more than 48 hours for Impute2 and Beagle, respectively. For the shorter chromosome i.e. ECA31, with about 150 000 SNPs, a complete run with FImpute was finished after 7 minutes, compared to 1 and 4 hours with Impute2 and Beagle.

**Table 1:** **Imputation accuracies on two chromosomes with three software programs**

**Software**	**ECA16**	**ECA31**
Impute2	0.927	0.920
Beagle	0.750	0.743
FImpute	0.774	0.772

The values indicate the concordance between true and imputed genotypes for two different chromosomes.

For the two programs that use population-wide LD, Beagle and Impute2, we further evaluated various imputation scenarios using different parameters settings on ECA31. For Beagle, we found that pre-phasing led to an increase in accuracy of about 3%, while addition of first-generation family information increased the accuracy only by 1% (Table 2). For Impute2, alternative settings of the SHAPEIT parameters were tested to identify optimal parameter settings for imputation of the whole genome (Table 3). Apart from accuracy, we also evaluated the number of imputed SNPs per individual that passed the genotype probability of 0.9. The tested parameters for SHAPEIT included pedigree information, recombination rate (rho), window-size, and N_e_. For Impute2, we also tested the length of the imputed interval, N_e_, and the impact of pre-phasing. Most of these changes did not have a major influence on imputation accuracy or the number of imputed SNPs passing the probability threshold (Table 3). Only the use of the default value for N_e_ (which is 15 000 for SHAPEIT and 20 000 for Impute2) increased the genotype accuracy from ~95% to ~97% but simultaneously decreased the number of SNPs passing the probability threshold from 130 000 to about 54 000 per individual. Based on these findings and the required time for file preparation and computation, we decided to use the default parameter settings, except for the use of a smaller N_e_ (=100) for imputation of the full genome datasets.

**Table 2:** **Imputation accuracies on chromosome ECA31 using Beagle with different parameter settings**

**Accuracy**	**Prephasing**	**Pedigree**
0.717	no	no
0.743	yes	no
0.753	yes	yes

Three different parameter settings for Beagle were evaluated; pre-phasing of the reference population was optionally performed in a separate step using Beagle; the accuracy was also determined with and without feeding pedigree data (duos and trios) into the program.

**Table 3:** **Imputation accuracies on chromosome ECA31 using Impute 2 with different parameter settings**

**Concordance**	**SNP number**	**N**_**e**_ **SHAPEIT**	**N**_**e**_ **Impute2**	**Pedigree**	**rho**	**Window size**	**Prephasing with SHAPEIT**	**Imputation intervall**
0.954	129 985	100	100	yes	0.0004^2^	2 Mb^2^	all horses	6 Mb^2^
0.950	128 845	100	100	yes^1^	0.0004^2^	2 Mb^2^	all horses	6 Mb^2^
0.953	130 103	100	100	yes^1^	0.0004^2^	2 Mb^2^	all horses	6 Mb^2^
0.950	128 501	100	100	no	0.0004^2^	2 Mb^2^	all horses	6 Mb^2^
0.954	130 422	100	100	yes	0.0004^2^	2 Mb^2^	all horses	whole ECA31
0.953	130 001	100	100	yes	0.0004^2^	0.5 Mb	all horses	6 Mb^2^
0.952	129 511	100	100	yes	0.01	2 Mb^2^	all horses	6 Mb^2^
0.953	129 544	15 000^2^	100	yes	0.0004^2^	2 Mb^2^	all horses	6 Mb^2^
0.971	54 295	100	20 000^2^	yes	0.0004^2^	2 Mb^2^	all horses	6 Mb^2^
0.956	128 135	100	100	yes	0.0004^2^	2 Mb^2^	test pop. only	6 Mb^2^

The concordance of true and imputed genotypes was calculated with different parameter settings for ECA31; the number of SNPs passing the probability threshold of 0.9 on average per animal is indicated in the second column; Rho: recombination rate in SHAPEIT; the parameters in the first row are the same parameters as in Table 1, except for the quality threshold of 0.9 which was set to 0; ^1^only in reference population; ^2^default value of the software.

### Genome-wide genotype imputation accuracy with Impute2

We then performed imputation for each chromosome separately in the 26 FM horses, using the same cross-validation design as before. We attempted to impute 13 127 080 SNPs in total. On average, 11 770 355 SNPs per horse passed the probability threshold of 0.9.

The overall genotype accuracy between experimental (determined by NGS) and imputed genotypes was 95.3%. The lowest accuracy (91.9%) was found for ECA12. All other chromosomes had imputation accuracies greater than 94.0%. Chromosome ECA14 had the highest accuracy, at 96.2% [See Additional file 2].

**Figure 2:**
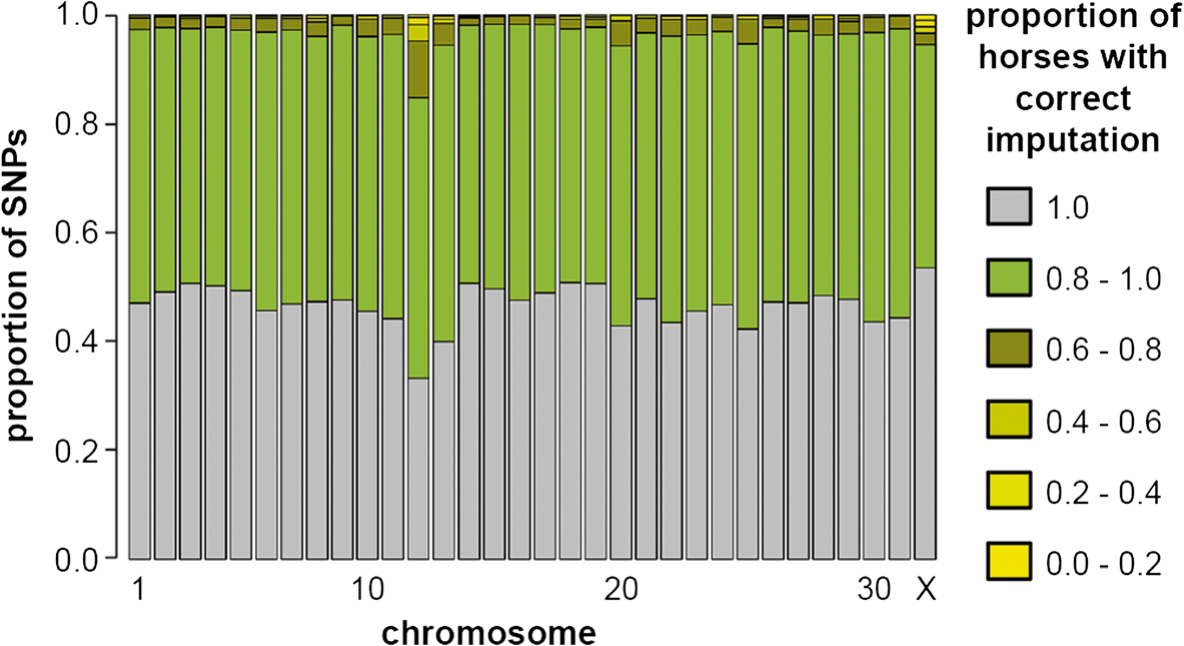
**Concordance between true and imputed genotypes.** The concordance was calculated in a cross-validation experiment comprising 26 FM horses for which 50 k SNP-chip and genome sequence data were available. The SNPs were divided into six concordance classes depending on the proportion of horses, which had correctly imputed genotypes. Roughly half of the SNPs showed perfect concordance between experimental and imputed genotypes and only very few SNPs had incorrect genotypes in more than 20% of the horses. Note the lower concordance of true and imputed SNPs on ECA12.

Around 50% of the SNPs were accurately imputed (meaning an accuracy of 100% over all horses) for each animal and the vast majority of the SNPs were correctly imputed in at least 80% of the horses (Figure 2).

### Individual imputation accuracy per horse

**Figure 3:**
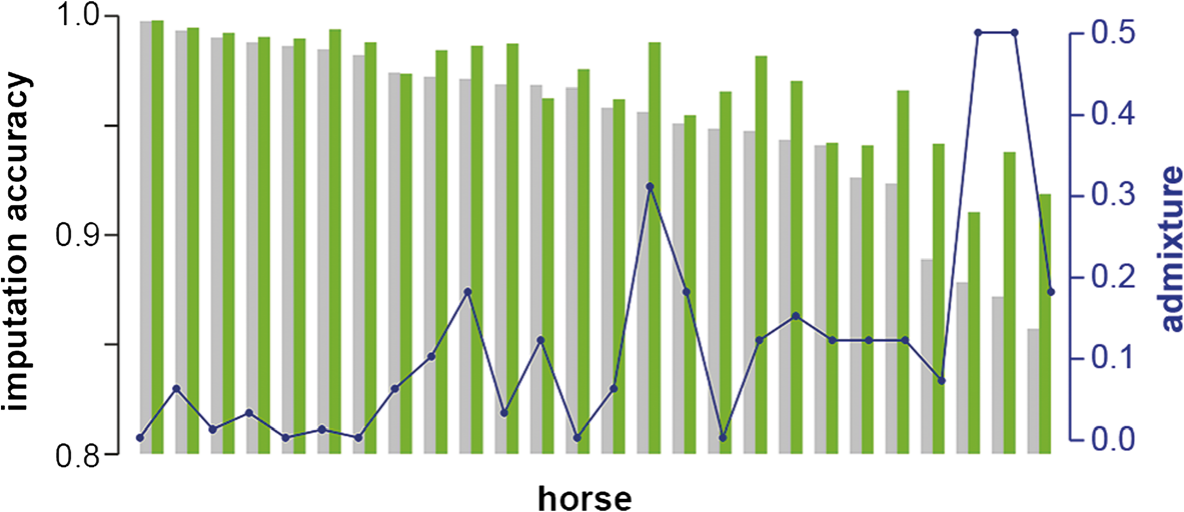
**Accuracy and admixture per horse.** The grey shaded bars show the accuracy per individual when only 28 FM horses were used as reference, the green bars show the accuracy when 14 Warmblood horses were added to the reference population and the blue dots show the Warmblood admixture of each evaluated FM horse.

The individual imputation accuracy was estimated based on results from imputing with Impute2 on ECA31. The accuracies per horse ranged from 85 to 99%. No difference in mean accuracy was found between males and females, while, the level of admixture of the horses was highly correlated with the individual imputation accuracy (r^2^ = −0.84) (Figure 3).

To account for the effect of admixture and to improve the overall imputation accuracy, we added genome sequence data of 14 Warmblood horses to our initial reference population of 28 FM horses. This led to an increase in the mean imputation accuracy from 95.3 to 96.8%. Individual accuracies increased for 24 of the 26 tested FM horses and ranged from 91 to 99% (Figure 3).

## Discussion

In this study, we evaluated the feasibility of imputing from 50 k SNP-chip data to almost 13 million SNPs in the FM horse breed. Initially, we obtained 50 k SNP-chip data on 1077 FM horses and genome sequences on 30 FM horses. The comparison of SNP-chip-derived genotypes versus sequence-derived genotypes provided an objective quality measurement for the NGS experiment and the variant calling pipeline. This comparison revealed that two horses had a poor genotype concordance between SNP-chip- and sequence-derived genotypes, which led to their exclusion from the analyses. The most probable reason why these two horses had such a low concordance was their low sequencing coverage. Subsequently, additional sequence reads for these horses were collected, which brought their genotype concordance to the same level as for all the others (data not shown), but this data was not used in the analyses presented here.

Here, we selected a total of 30 representative FM horses for re-sequencing. These horses explain a large fraction of the genetic variance of the population and therefore maximize the correct imputation of causal mutations [9,14]. Thus, we can expect to obtain most of the common variants but also variants that differ between the different stallion lineages. In addition, several phenotypes (e.g. linear description and conformation traits) of interest are covered by the sequenced animals, so that causal mutations for these traits should be included in our data. However, despite the optimal selection of representative individuals, it is possible that recent and very rare mutations are not contained in our limited dataset.

We evaluated imputation accuracy using three software programs. We found that Impute2 had the highest imputation accuracy. This software has also been found to outperform Beagle, fastPhase, and FImpute in cattle [10,11]. Several factors may explain the difference in performance between imputation softwares, including method- and population-based differences (e.g. the reconstruction of haplotypes, the extent of LD and the size of the reference population). The greater accuracy of Impute2 in our dataset might be due to the extended LD that is present in the FM breed [36], which allows for a better definition of long-range haplotypes. As mentioned before, Impute2 reconstructs haplotypes based on SNP information of the reference and test population, which becomes especially useful for small reference populations. Therefore, the difference between the two population-based methods (Impute2 and Beagle) is likely a result of the small reference population used [9]. Increasing the size of the reference population should result in the convergence of the accuracies of these two methods [9]. Although we assumed that all discrepancies between the imputed and sequenced-derived genotypes were due to imputation errors, it is clear that discrepancies could also be caused by sequencing errors.

Imputation with different parameter settings for Beagle showed that pre-phasing and inclusion of first-generation pedigree information (duos and trios) increased the accuracy of genotype imputation, with pre-phasing having a greater impact than including the pedigree information. Using Impute2, pre-phasing the test population only slightly improved the genotype accuracies (Table 3). Nevertheless, we recommend the data to be pre-phased and to include first-generation pedigree information in order to increase computational efficiency and haplotype reconstruction. We also explored the effect of different N_e_ settings in more detail. Using the default setting (N_e_ = 20,000) resulted in highly accurate genotypes but more than 60% of the SNPs did not pass the probability threshold of 0.9. Therefore, we used current N_e_ estimates for the FM breed (Ne = 100) in the final genotype imputation.

We showed that 50 k genotypes could be directly imputed to sequence level genotypes. In cattle, SNP imputation is usually performed in multiple steps. 50 k SNP-chip data are imputed to HD data and then to sequence level [10]. The low N_e_ and the high genetic relatedness between FM horses also allow low-frequency alleles to be directly imputed from 50 k to sequence level genotypes with reasonable accuracy. However, with the upcoming release of an HD SNP-chip for horses, we expect that the imputation accuracy for our FM horse population can be further improved, by using HD genotypes for intermediate genotype imputation [37].

Most of the SNPs had a high accuracy. Compared to the other chromosomes, the accuracy was markedly reduced for ECA12. We suspect that SNPs on this chromosome may have incorrect positions in the reference genome EquCab2.0 or contain errors in sequencing calls, since this chromosome also showed a low concordance between SNP-chip- and sequence-derived genotypes. Imperfect concordance between NGS data and EquCab2.0 is not a completely unknown phenomenon [38].

In this study, the number of animals in the reference population was small. Thus, we designed a cross-validation scheme to measure the imputation accuracy for each horse. Despite the optimal choice of representative horses and the high level of LD within the FM breed, we observed high variations in genotype imputation accuracies between horses. We showed that the major factor causing the variation between horses was the level of admixture with introgressed Warmblood horses. Therefore, we expect that sequence level genotype imputation will result in greater imputation accuracies in closed populations than in highly admixed populations, especially when the number of sequenced animals and relatives is limited [9]. For the FM breed, we increased the genotype accuracy per horse by including Warmblood horses in the reference population.

## Conclusions

Our data show that imputation from 50 k SNP-chip data to 13 million SNPs with 95% accuracy is feasible in the FM horse breed. Impute2 was the best software for imputation in our dataset and the inclusion of additional Warmblood reference sequences increased the accuracy of imputation furthermore.

## Additional files

Additional file 1.
**Summary statistics of next-generation sequencing.** In this file, some basic summary statistics of NGS and genotype concordance with the SNP-chip data per horse are provided. Additional file 2.
**Number of SNPs used for imputation.** For each chromosome, the number of SNPs and the concordance of imputation are listed in this table.
